# 

**Published:** 1910-06

**Authors:** 


### BOOKS RECEIVED.

Spondylotherapy. Spinal concussion and the application of other methods to the spine in the treatment of disease. By Albert Abrams, A.M., M.D., Consulting Physician to the Mount Zion and French Hospitals, San Francisco. 12 mo., pp. 417. Illustrated. San Francisco: The Philopolis Press. 1910.

Diseases of the Eye. By G. E. deSchweinitz, A.M., M.D., Professor of Ophthalmology in the University of Pennsylvania. Sixth edition. Octavo, pp. 945. Illustrated. Philadelphia and London. W. B. Saunders Co. 1910. (Cloth, $5.00.)

Transactions of the American Urological Association. Eighth annual meeting held at Atlantic City, N. J., June 7 and 8, 1909. Edited by Charles Greene Cumston, M.D., Boston.

An Index of Symptoms with Diagnostic Methods. By Ralph W. Leftwich, M.D., Late Assistant Physician to the East London Children's Hospital. Fourth edition. 12 mo, pp. 451. New York: William Wood & Co. 1910. (Cloth, $2.25.)

Physiology and Pathology of the Semicircular Canals. By Adolph E. Ibershoff, M.D. 12 mo, with 8 illustrations. New York: Paul B. Hoeber. 1910. (Cloth, $1.00.)

The Pathology of the Living and Other Essays. By B. G. A. Moynihan, M.S. (London), F.R.C.S., Honorary Surgeon to Leeds General Infirmary; Professor of Clinical Surgery at the University of Leeds, England. 12 mo, of 260 pages. Philadelphia and London: W. B. Saunders Company, 1910. (Cloth, $2.00 net.)

Medical Electricity and Röntgen Rays. By Sinclair Tousey, A.M., M.D., Consulting Surgeon to St. Bartholomew's Clinic, New York. Octavo of 1116 pages, with 750 illustrations, 16 in colors. Philadelphia and London: W. B. Saunders Company, 1910. (Cloth, $7.00; half morocco, $8.50, net price.)

Pulmonary Tuberculosis and Its Complications. By Sherman G. Bonney, M.D., Professor of Medicine, Denver and Gross College of Medicine, Denver. Octavo of 955 pages, with 243 original illustrations, including 31 in colors and 73 x-ray photographs. Philadelphia and London. W. B. Saunders Company. 1910. (Cloth, $7.00 net; half morocco, $8.50, net prices.)

Surgical After-Treatment. By L. R. G. Crandon, A.M., M.D. Assistant in Surgery at Harvard Medical School. Octavo of 803 pages, with 265 original illustrations. Philadelphia and London: W. B. Saunders Company, 1910. (Cloth, $6.00; half-morocco, $7.50 net prices.)

The Medical-Epitome Series. Diseases of the Skin. A Manual for Students and Practitioners. By Alfred Schalek, M.D., Professor of Dermatology, University of Nebraska. Second edition. 12 mo, 255 pages, with 47 engravings. Lea & Febiger, Publishers, Philadelphia and New York, 1910. (Cloth, $1.00, net.)

